# Assembly, Assessment, and Availability of *De novo* Generated Eukaryotic Transcriptomes

**DOI:** 10.3389/fgene.2015.00361

**Published:** 2016-01-11

**Authors:** Joanna Moreton, Abril Izquierdo, Richard D. Emes

**Affiliations:** ^1^Advanced Data Analysis Centre, Sutton Bonington Campus, University of NottinghamLeicestershire, UK; ^2^School of Veterinary Medicine and Science, Sutton Bonington Campus, University of NottinghamLeicestershire, UK

**Keywords:** *de novo* transcriptome assembly, high-throughput sequencing, assessment, availability, annotation

## Abstract

*De novo* assembly of a complete transcriptome without the need for a guiding reference genome is attractive, particularly where the cost and complexity of generating a eukaryote genome is prohibitive. The transcriptome should not however be seen as just a quick and cheap alternative to building a complete genome. Transcriptomics allows the understanding and comparison of spatial and temporal samples within an organism, and allows surveying of multiple individuals or closely related species. *De novo* assembly in theory allows the building of a complete transcriptome without any prior knowledge of the genome. It also allows the discovery of alternate splice forms of coding RNAs and also non-coding RNAs, which are often missed by proteomic approaches, or are incompletely annotated in genome studies. The limitations of the method are that the generation of a truly complete assembly is unlikely, and so we require some methods for the assessment of the quality and appropriateness of a generated transcriptome. Whilst no single consensus pipeline or tool is agreed as optimal, various algorithms, and easy to use software do exist making transcriptome generation a more common approach. With this expansion of data, questions still exist relating to how do we make these datasets fully discoverable, comparable and most useful to understand complex biological systems?

## Introduction

It is desirable to fully understand the complexity of an organism and the diversity of cell types arising from a single genome, or to compare the compliment of genes between evolutionary groups. This requires a capability to view and catalog the changes in gene expression of a cell or tissue. The transcriptome is the complete set of transcripts (RNA molecules) within a cell including protein-coding and non-coding RNAs. Additionally, the transcriptome encompasses all alternative splice forms, alternatively polyadenylated, and RNA-edited transcripts. Together, these reflect the genes that are actively expressed in a particular tissue (Grobe et al., 2002; Lu et al., 2013). Understanding the complete transcriptome is a technical challenge requiring technologies for capturing an accurate representation of the RNA in a cell or tissue. The dominant technology for the assessment of gene expression was microarrays which use printed or synthesized probes corresponding to mRNAs (Fu et al., 2009). Whilst these technologies are robust and offer a more mature framework for data analysis, they require an already annotated complete genome to design the probes. Microarrays are also limited by inaccurate hybridization of sequences to probes, which is difficult to model and hence account for (Wang et al., 2009; Compeau et al., 2011). In the case of model organisms, microarrays are still hugely useful to measure and compare gene expression. However, where high quality **annotation** and appropriate arrays do not exist, DNA sequencing offers the best method to understand the transcriptome. With the advent of Next Generation Sequencing (NGS) technologies and improved extraction methods to accurately purify RNA from smaller amounts of tissue or even single cells (Islam et al., 2011), the possibility to catalog and measure gene expression from a wider range of organisms has become possible.

KEY CONCEPT 1 **Annotation**

The process of assigning functional information to transcripts, such as gene ontology terms, in order to characterize the sequences and allow understanding of the system studied.

**Figure 1:**
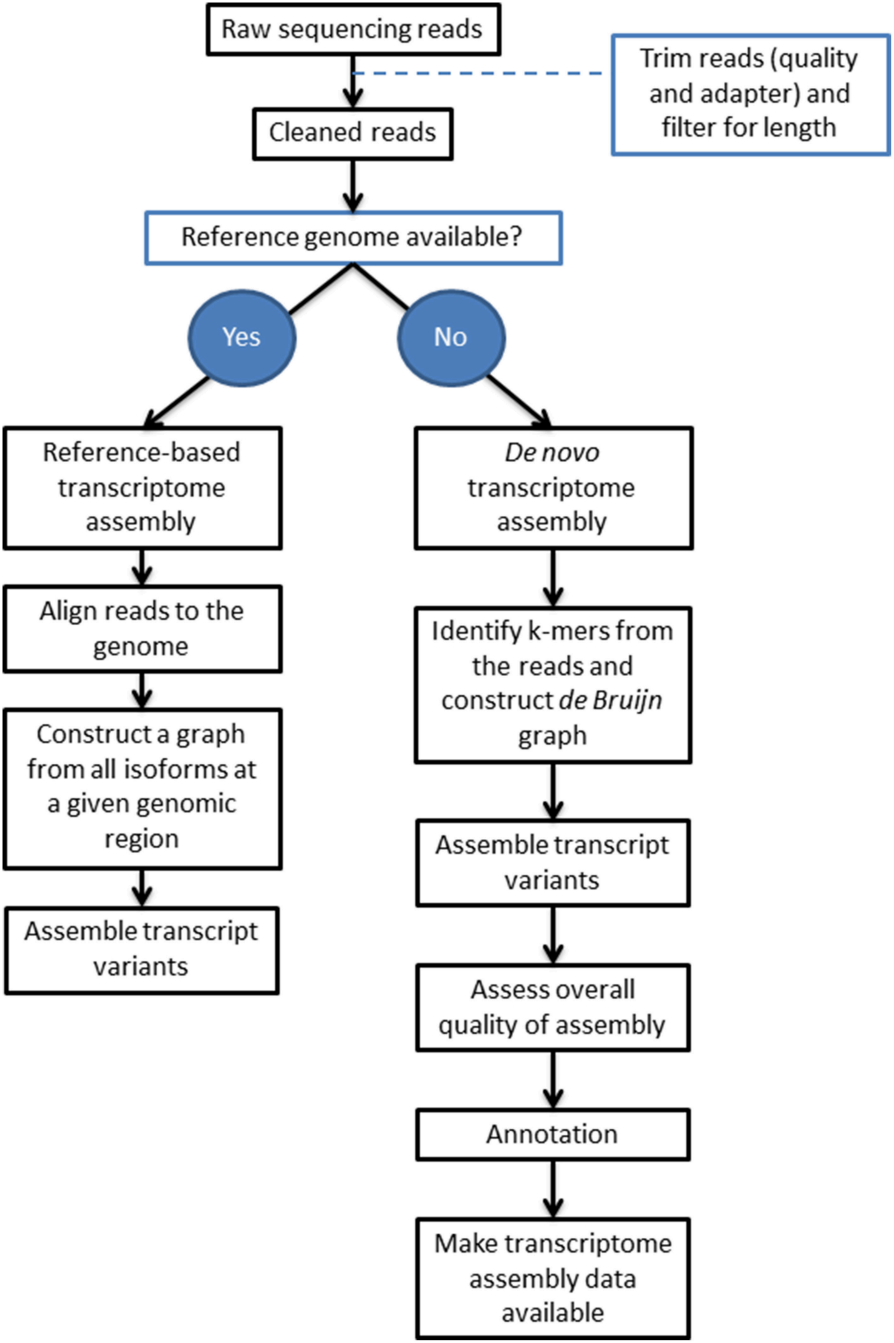
**An overview of the two transcriptome assembly pipelines**. The key parts of two transcriptome assembly pipelines are shown depending on whether a reference genome is available. This review is focused on *de novo* transcriptome assembly; more information on the pipeline for reference-based transcriptome assembly can be found in review papers such as Martin and Wang (2011).

Transcriptome assembly is the process of identifying transcripts and their variants that are expressed in a determined sample (Lu et al., 2013). The simple premise is to reconstruct the complete sequences of all transcripts in the transcriptome. It is uncommon to achieve this in practice as most of the time the sequencing depth is not sufficient to cover all full-length transcripts, particularly the ones of low abundance. A transcriptome is therefore a set of contiguous (contig) sequences that represent transcript regions (Li et al., 2014). Generally the strategies for transcriptome assembly fall into two categories: reference-based and *de novo* (Figure 1), although a combination of both can be used (Chen et al., 2011; Garber et al., 2011; Martin and Wang, 2011; Haas et al., 2013; Lu et al., 2013). Whilst a comprehensive set of tools is unrealistic, we have compiled a set of commonly used, freely available tools for *de novo* assembly and assessment (Supplementary Table 1).

## Transcriptome assembly methods

### Reference-based transcriptome assembly method

**Reference-based transcriptome assembly** is widely used when a model organism, with a sequenced genome for the target transcriptome, is accessible. Thus, the transcriptome is reconstructed by mapping to previously known sequences (Martin and Wang, 2011). The short reads are aligned to the reference genome allowing the overlapping regions to be assembled into transcripts. Where a good quality reference exists, the reference-based strategy is highly sensitive and it has become the basic method for many RNA sequencing (RNA-seq) studies. However, the accuracy of reference-based transcriptome assembly depends on correct read alignment, and issues such as alternative splicing and sequencing errors increase the difficulty of this task (Grabherr et al., 2011). In a referenced-based assembly approach, the sequence reads are aligned to the genome using a tool such as TopHat2 (Kim et al., 2013), which takes splicing into consideration. This is necessary as copies of mature spliced RNA have been sequenced, but these need to be mapped to a genome containing introns. All alternative splicing events are then captured in a graph for each given locus. Different paths are traversed in the graph to find transcript variants (Martin and Wang, 2011). Two transcriptome assemblers that are commonly used for graph building and traversal are Cufflinks (Trapnell et al., 2010) and Scripture (Guttman et al., 2010). The computational requirements of reference-based transcriptome assembly are significantly less compared to ***de novo* transcriptome assembly**. Furthermore, the presence of artifacts or sequencing contamination does not represent a major issue since these can often be resolved when aligning the reads to the genome. However, the quality of the results depends largely on the quality of the genome model used.

KEY CONCEPT 2 **Reference-based transcriptome assembly**

A method which is used to reconstruct transcript sequences by aligning RNA sequencing reads to a reference genome.

KEY CONCEPT 3 ***De novo* transcriptome assembly**

A process by which overlapping RNA sequencing reads are combined without a reference genome to reconstruct transcript sequences.

The transcriptome assembly can also be complicated by reads that align to multiple sites in the genome; these are known as multi-mapped reads. This problem is increased if the reads are short, therefore large complex transcriptomes are not easily assembled from very short reads (Martin and Wang, 2011). If there is insufficient unique information in the read sequences, then it is difficult to assign the reads to the correct location during alignment to the reference genome. If multi-mapped reads are discarded, then information for non-unique regions will be lost including gene families where gene sequences can be highly similar (Robert and Watson, 2015). If they are retained, it can be a challenge to accurately estimate gene or transcript abundances (Patro et al., 2014). Recently, Robert and Watson (2015) proposed a method for dealing with multi-mapped reads. They suggest taking all of the reads that cannot not be aligned to a unique gene and instead allocating them to a “multi-mapped group.” These groups are determined from the RNA-seq data rather than relying on existing annotation. By performing differential expression analysis on multi-mapped gene groups, rather than individual genes, important biological information can be examined that would have otherwise been filtered out (Robert and Watson, 2015).

Once reads are mapped and transcripts are identified, there are tools that can be used to quantitate gene expression such as Cufflinks (Trapnell et al., 2010), DESeq2 (Love et al., 2014), or EdgeR (Robinson et al., 2010). Thus, for organisms with an accurate, complete and well annotated genome, the measurement of genes expressed in a sample is becoming commonplace with robust methods for mapping transcript fragments to the genome and measuring the transcriptome content. However, where an annotated genome does not exist, or the number of alternate transcript isoforms is high, the problem of generating an accurate representation of the complete transcriptome remains. It is in these situations that *de novo* transcriptome assembly is particularly attractive as it provides an alternative option for assessing a non-model transcriptome (Zhao et al., 2011). *De novo* transcriptome assembly works without a reference to attempt to directly reconstruct overlapping reads into transcripts (Grabherr et al., 2011; Martin and Wang, 2011; Clarke et al., 2013; Lu et al., 2013). The complexities of this approach make it more computationally demanding, however a range of software tools exist including Oases (Schulz et al., 2012), Trans-ABySS (Robertson et al., 2010), MIRA (Chevreux et al., 2004), and Trinity (Grabherr et al., 2011). Several studies have been carried out to evaluate the execution of transcript assemblers (e.g., Clarke et al., 2013), and although they all differ in performance, currently there is no single transcriptome assembler categorized to be the best option for every condition (Grabherr et al., 2011; Clarke et al., 2013; Góngora-Castillo and Buell, 2013; Lu et al., 2013). With these specialist comparisons of performance available, it is not the objective of this review to describe nuances of different approaches or to promote a single method as optimal. In many cases the use of multiple approaches and subsequent merging of assemblies to generate a consensus single or set of assemblies might be appropriate. For example, incorporating sequences from different assemblers and parameters to generate a consensus transcriptome, by using transcripts present in multiple original transcriptome assemblies (Moreton et al., 2014).

### *De novo* transcriptome assembly method

**Figure 2:**
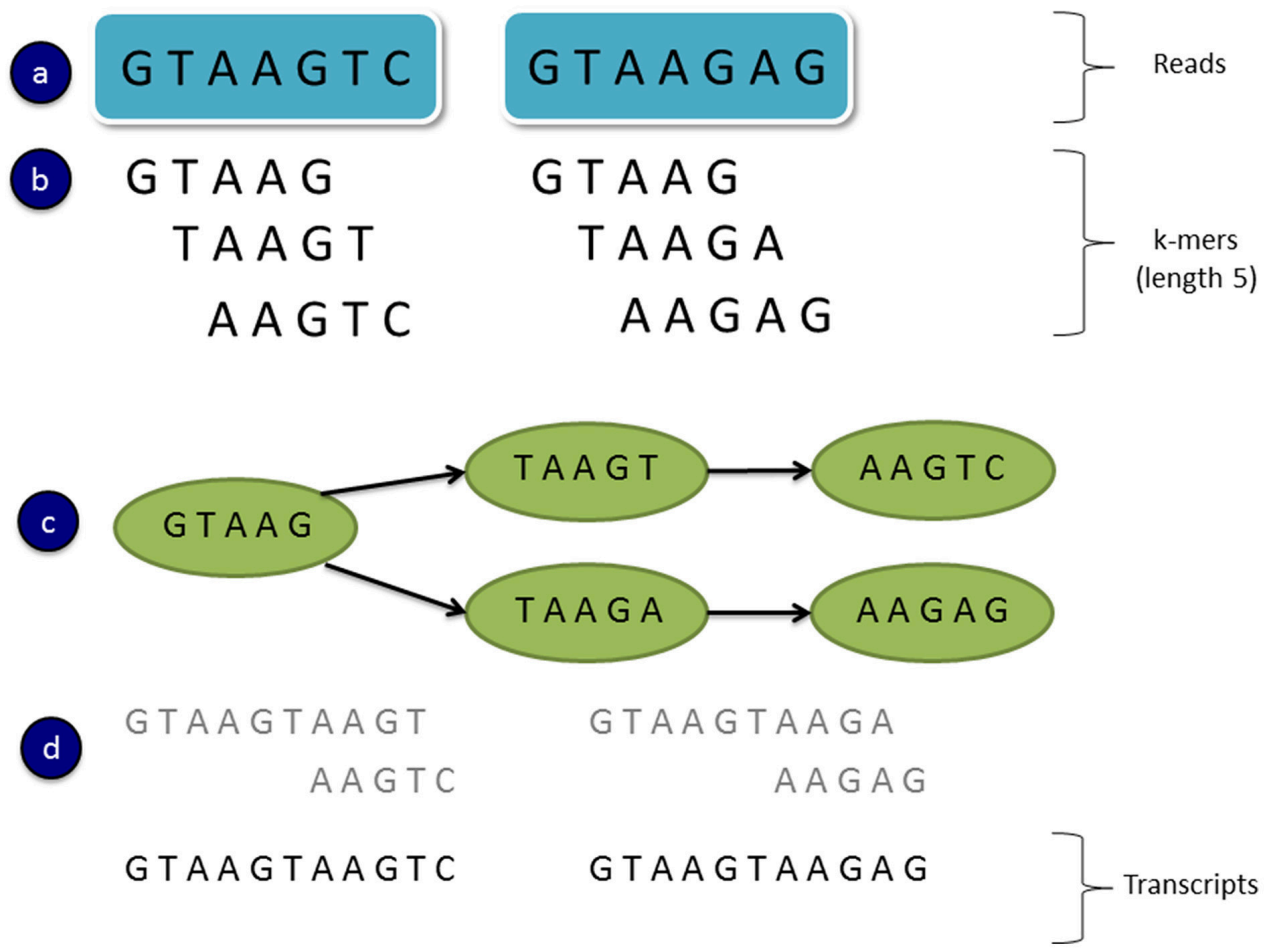
**An example of a simple *de Bruijn* graph. (A)** Read sequences **(B)** All subsequence k-mers of length 5 from the reads **(C)** A *de Bruijn* graph constructed from unique k-mers as the nodes and overlapping k-mers connected by edges (a k-mer shifted by one base overlaps another k-mer by k-1 bases) **(D)** Assembled transcripts by traversing the two paths in the graph.

*De novo* transcriptome assemblers commonly use a strategy which involves constructing *de Bruijn* graphs (e.g., Grabherr et al., 2011; Schulz et al., 2012). In this approach all subsequences of length k are found in the reads and these are known as “**k-mers**.” A *de Bruijn* graph is created using all unique k-mers as nodes, with connecting edges representing immediately overlapping k-mers (Figure 2). That is if a k-mer substring is shifted by one sequence base, and it overlaps another k-mer (by k-1 bases), then an edge is drawn between the nodes associated with those k-mers (Martin and Wang, 2011). A linear chain of k-mer nodes is compressed into a single node where possible (where the two nodes are joined by a single unique edge). Transcript variants can then be assembled by traversing the paths of the graph. Figure 2 shows a toy example of a *de Bruijn* graph constructed from two 7 bp sequence reads and k-mers of length 5. In this example two paths can be found from the graph representing two possible transcript isoforms.

KEY CONCEPT 4 **k-mers**

A subsequence of specified length k. They are often used by *de novo* assemblers to allow sequence information to be compacted, which makes reconstruction of transcripts easier computationally.

Before the introduction of *de Bruijn* graphs, assemblers used the overlap-layout-consensus algorithm where overlap information between read sequences is added to a mathematical graph to find a consensus sequence (Li et al., 2012b). In this strategy, each graph node corresponds to a read and if two reads overlap, their nodes are joined by an edge on the graph. The overlap-layout-consensus alignment step is computationally intensive when assembling a huge number of short reads, so a *de Bruijn* graph algorithm is preferred for generating *de novo* assemblies. By compacting the sequence information into k-mers, the graph theory method for finding a path in the graph becomes easier computationally (Pevzner et al., 2001; Li et al., 2012b). One disadvantage in using the *de Bruijn* graph approach is the generation of misassembled contigs which occurs because of the use of k-mers (Clarke et al., 2013). If two transcripts from different genes have the same k-mer sequence they could be erroneously connected. The computational proficiency of the *de Bruijn* graph strategy is clearly beneficial, but it is an ongoing problem to balance this with assembly accuracy (Clarke et al., 2013).

There are a number of difficulties that are encountered by the *de novo* transcriptome assembly strategy. For example, it is challenging to discriminate between transcript variants that are produced from processes such as alternative splicing or sequences transcribed from paralogous genes (Grabherr et al., 2011; Vijay et al., 2013). These sorts of sequences will share k-mer sequences and hence it is difficult to tease them apart into separate transcripts. Software tools have been designed to distinguish transcript variants using paired-end read data and read coverage (Góngora-Castillo and Buell, 2013). For instance, the Trinity assembler (Grabherr et al., 2011) reconstructs alternatively spliced transcripts and paralogous sequences by clustering overlapping contigs and generating a *de Bruijn* graph for each cluster of sequences independently. These graphs are then supplemented with the read and paired-end information to generate all possible transcript variants. Despite the challenges, the transcriptomes of many different organisms have been assembled using the *de novo* approach (e.g., Kumar and Blaxter, 2010; Robertson et al., 2010; Zhao et al., 2011; Price et al., 2015). These complexities are additionally compounded when mixed samples are included, for example in pathogen and host, or when transcripts may not form distinct entities due to dense or overlapping transcripts, as seen in prokaryote organisms. In the case of bacterial *de novo* assembly, tools such as Rockhopper (McClure et al., 2013; Tjaden, 2015) have been specifically developed.

## Assessment of generated *De novo* assemblies

Whilst a number of studies have focused on transcriptome assembly, the assessment of the overall quality of the derived assemblies is less well defined. A number of different measures are commonly used to evaluate assembled transcriptomes. Commonly used metrics when there is no close reference include the number of contigs (transcripts) assembled, summed contig length, mean transcript length, N50 value, and the proportion of reads that could be mapped back to the assembled transcripts (RMBT; e.g., Zhao et al., 2011). These measures can be used to compare and select optimal assemblies, for example the N50 value can be maximized whilst keeping the total assembly length as long as possible (Zerbino, 2010). It is also important to consider the time taken to generate the assemblies (Kumar and Blaxter, 2010). When reference sequences of closely related species are available, the assembled contigs can be compared using a sequence similarity tool such as BLAST (McGinnis and Madden, 2004) to assess the validity of the assembly (e.g., Arun-Chinnappa and McCurdy, 2015; Ghaffari et al., 2015). However, this approach is biased by the appropriateness of the choice of related species for comparison and will be biased toward available “model” genomes.

Assessment of the completeness of an assembled transcriptome is more problematic. This is due to the impossibility of knowing *a priori* what the complete transcriptome for a previously unsequenced cell, or collection of cells, at a particular time point is. However, the theoretical completeness can also be assessed, using methods to determine the assembly of transcripts that are expected to be present in all cells at all times, such as the Core Eukaryotic Genes Mapping Approach (CEGMA) tool by Parra et al. (2007). Although not developed specifically for this purpose, many studies have used this approach to determine if a collection of newly assembled transcripts encode one or more of a set of core genes conserved across a wide range of eukaryotic species, thus providing a percentage “completeness” score (e.g., Chauhan et al., 2014; Moreton et al., 2014; Frías-López et al., 2015; Powell et al., 2015; Price et al., 2015). A recent web-based tool “TRUFA,” developed by Kornobis et al. (2015), incorporates CEGMA into its pipeline as part of the assessment stage of *de novo* assemblies. As of May 2015 CEGMA is no longer being supported, however a new tool “BUSCO” has been published by Simão et al. (2015), to assess assembly and annotation completeness using sets of Benchmarking Universal Single-Copy Orthologs (BUSCO), selected from OrthoDB (Kriventseva et al., 2015). When comparing the completeness of genome assemblies and gene sets across 40 species, the BUSCO assessments were more consistent than CEGMA, the run-times were much faster and the software can also be used to assess gene sets and transcriptomes (Simão et al., 2015).

Some authors have suggested that evaluation measures such as N50 might be misleading and uninformative for evaluating transcriptome assemblies (e.g., O'Neil and Emrich, 2013; Li et al., 2014; Chen et al., 2015). For example, Chen et al. (2015) found that the transcriptome assemblies with the highest N50 values, did not make a significant contribution to the best assembled transcript set based on coding potential. Li et al. (2014) developed the “DETONATE” (DE novo TranscriptOme rNa-seq Assembly with or without the Truth Evaluation) software, which includes both reference-free (RSEM-EVAL) and reference-based (REF-EVAL) methods. The reference-free approach is based on a probabilistic model that uses only the read and assembly data. When reference transcripts are available, the REF-EVAL component can be used to generate scores based on different reference-based measures. DETONATE is currently only designed to evaluate assemblies generated from Illumina data, although there are plans to update the package to handle data from other sequencing platforms. O'Neil and Emrich (2013) assessed a number of metrics for *de novo* transcriptome assemblies including unique annotations and “ortholog hit ratio” from their earlier work (O‘Neil et al., 2010). The correlation between the REF-EVAL score and the ortholog hit ratio measure was found to be low, although the number of unique proteins matched had good correlation to REF-EVAL (Li et al., 2014).

There are a number of errors that can occur in *de novo* transcriptome assembly, for example two transcripts may be combined into a single false chimeric transcript, or contigs might be incomplete or mis-assembled (Smith-Unna et al., 2015). These errors can be detected using read evidence. The *TransRate* tool (Smith-Unna et al., 2015) aligns the paired-end reads that were used to generate the assembly, back to the assembled contigs. The alignments are then evaluated and each contig is assigned a score based on properties such as how well the nucleotides in the aligned reads matched to the assembled contigs, the coverage of the contig nucleotides, and the order of the contig nucleotides based on the paired-end read orientations. *TransRate* also calculates an assembly score which is generated from the individual contig scores, and the proportion of input reads that were incorporated into the *de novo* assembly. As mentioned before, RSEM-EVAL is another reference-free evaluation method; however it does not focus on the evaluation of individual contigs. The RSEM-EVAL tool is also limited to assemblies generated from Illumina data, but *TransRate* is not restricted in this way. The *TransRate* tool is also useful because it allows the filtration of individual contigs based on their scores. Furthermore, the authors used 155 previously published *de novo* assemblies in a meta-analysis to allow users to analyze their assemblies in comparison with others. In summary, assembly assessments are essential and will be increasingly important for evaluation of new methods, or in the combination of assemblies as part of optimization strategies.

## Annotation of transcriptome assembly

Annotation of function is required to characterize transcripts and allow understanding of the system studied. Most approaches to annotation of protein coding transcripts use one or more homology based approaches to identify related sequences of known function, and hence transfer this annotation to the new transcript (Emes, 2008). There are however limitations to these approaches. The problem of transfer of inappropriate or inaccurate annotation from one dataset to another, leading to the propagation of annotation error, is the most concerning. A preferred method is the use of protein domain architecture to drive the annotation. Searching for conserved domains using hidden Markov model search tools, such as HMMER3 (Finn et al., 2011), is a relatively simple process. These tools search comprehensive libraries of domains such as Pfam (Finn et al., 2014) or InterPro (Mitchell et al., 2015). Databases such as Pfam2GO, from the gene ontology consortium (Gene Ontology Consortium, 2015), allow the domain content to generate restricted descriptors of each transcript. Pipeline tools to automate this process using both sequence similarity and domain composition, such as the Trinotate pipeline (https://trinotate.github.io/), are available but are currently relatively slow or computationally intense to use. Another consideration for the annotation process is searching for repeat elements using programs such as RepeatMasker (http://www.repeatmasker.org) or the Tandem Repeats Finder (Benson, 1999). For example, RepeatMasker can be used with the Repbase database (Bao et al., 2015) to identify transposable elements and other types of repeats (Gillard et al., 2014; Kumar et al., 2014; Cokus et al., 2015; Richardson and Sherman, 2015).

## *De novo* transcriptome assembly availability

Whilst most journals require raw sequencing reads to be made publicly available in a database such as the Sequence Read Archive (SRA; Kodama et al., 2012), often the assembled transcripts and annotations are not made available. This results in lack of clarity and wasted effort to redo the analysis. The SRA is part of the International Nucleotide Sequence Database Collaboration (Kodama et al., 2012). This repository is available at the National Center for Biotechnology Information (NCBI, www.ncbi.nlm.nih.gov/sra), European Bioinformatics Institute (EBI, www.ebi.ac.uk/ena), and DNA Data Bank of Japan (DDBJ, http://trace.ddbj.nig.ac.jp/dra). There are support pages and handbooks to help with submitting data, and these are available at the NCBI, EBI, and DDBJ websites. As well as raw sequence data, alignment files in BAM (Li et al., 2009) format can also be submitted to the SRA. With reducing costs of sequencing and availability of software for transcriptome assembly, the making of transcriptome assembly open and available is a key problem in bioinformatics. Often generic genome browsers are difficult to set up and are not well-suited for transcriptome data (Jones and Blaxter, 2013), and so a number of software solutions to host and visualize transcriptome assemblies have been developed. Jones and Blaxter (2013) developed the web application “afterParty” which enables users to make a transcriptome publicly available. The application can take as input either Roche 454 reads, or assembled contigs (putative transcripts) from any platform. If raw 454 sequencing reads are used as an input, then afterParty can assemble them using MIRA (Chevreux et al., 2004) and then annotate the resulting contigs using BLASTX (Altschul et al., 1997), UniProt (Uniprot Consortium, 2012), and InterProScan (Zdobnov and Apweiler, 2001). In the other afterParty workflows, contigs generated by the user from any sequencing platform can be uploaded with or without annotation. AfterParty can also be used to browse transcriptomes and visualize data sets in a web browser. For example, all contigs with annotation matching a particular search term can be used to generate a scatter plot of GC content against coverage in a comparison to the full assembly (Jones and Blaxter, 2013). Different contig sets, chart types, and displays can be selected. In addition to filtering by annotation, a DNA or protein sequence can be used to find contigs with sequence similarity. The contigs can also be searched by properties such as length, quality, coverage, and GC content. A number of studies have already used the afterParty website as a means of hosting and distributing transcriptome data (e.g., Heitlinger et al., 2014; Short et al., 2014; McTaggart et al., 2015). For users running afterParty locally, the source code, and dependencies can be installed. However, the more convenient method would be to use the virtual disk image (available on GitHub), which contains all the required dependencies to run the software using a virtual machine. Alternatively, afterParty is also available through a public server.

RNAbrowse is an alternative package with a web interface that can be used to store and visualize *de novo* transcriptome data (Mariette et al., 2014). It is based on the BioMart (Smedley et al., 2015) software and in addition to the web interface it includes a command line tool for administration which requires a unix server and MySQL database. The project introduction page of the web interface contains useful information such as the software and parameters used to generate the alignment, annotation, assembly, and variant analysis. The contig and variant overview pages show general statistics and related figures such as a bar chart of contig length distribution. There is a blast query form to search the contigs using an input sequence, and the BioMart search page can also be used to filter the data based on criteria such as contig name, length, or annotation. In the sequence view, the longest open reading frame can be identified. It is also possible to view the sequences and annotations in JBrowse (Skinner et al., 2009) and compare read coverage between samples in the contig depth view. The figures produced using the interface can be easily printed or downloaded and there is also a dedicated download page to enable users to save some or all of the data (Mariette et al., 2014). In its simplest form, RNAbrowse can be set up using the assembled contig sequences (FASTA format) alongside the annotation and alignment files. Again, installation requires a number of prerequisite tools and the setup process can be quite time consuming (Mariette et al., 2014). This may therefore be better attempted in collaboration with a bioinformatics group or local support. However, there is a project website with lots of information about RNAbrowse including guides, demonstrations, example datasets and a configuration file template for larger projects. Different schedulers can also be selected to address any time issues (Mariette et al., 2014). As an example of a practical use, RNAbrowse has been used to display and distribute beech tree *de novo* transcriptome data (Lesur et al., 2015).

Apart from more complete packages such as afterParty and RNAbrowse, there are limited tools with web interfaces that are available for analysis of transcriptome data. CBrowse (Li et al., 2012a) is a web browser which takes assembled contig sequences and BAM/SAM alignment files as input, and enables the user to identify polymorphisms and view the contigs in the web interface. Its focus is not on annotation, however CBrowse can be used to disseminate assembled transcriptome data (Li et al., 2012a). As a less permanent solution, some research groups have used individual online resources to make their data available. For example, Aya et al. (2015) developed a transcriptome database as a public web resource for downloading and browsing fern *de novo* transcriptome assembly data, where both BLAST and keyword searches can be performed. Another research group released their axolotl read and transcriptome assembly data on a website with a keyword search facility (Stewart et al., 2013). However, the risk of non-specialist solutions is that repositories are not maintained or, with the movement of personnel, that the skill to maintain repositories is lost. As an interim solution, we and others have simply made transcriptome assembly data available to download by partnering with appropriate journals (Moreton et al., 2014; Ghaffari et al., 2015). Given these considerations, and the enhanced ability to query, filter and visualize transcriptome data, tools like afterParty, and RNAbrowse make the most ideal options.

## Conclusion

As the desire to catalog and compare the varied transcriptomes of complex organisms continues, *de novo* transcriptome assembly is an important tool in the bioinformatician's arsenal. Whilst rapid progress in single molecule sequencing is being made, it is currently not mature and so assembly, annotation and assessment of transcriptomes from relatively short reads will continue to be essential. To make these methods truly useful, assemblies that are accurately assembled and annotated are essential, but also the availability and openness of assembled transcriptomes not simply raw data must become expected practice.

## Author contributions

JM, AI, and RE wrote the paper, prepared figures, and reviewed drafts of the paper.

### Conflict of interest statement

The authors declare that the research was conducted in the absence of any commercial or financial relationships that could be construed as a potential conflict of interest.
