# Supplementary material for: Assembly, Assessment, and Availability of De novo Generated Eukaryotic Transcriptomes
Source: Front Genet. 2016 Jan 11;6:361. doi: 10.3389/fgene.2015.00361 (PMC4707302; doi:10.3389/fgene.2015.00361)
Supplement: Supplementary file 1 [file Table1.DOCX]

Supplementary Material

Assembly, assessment and availability of *de novo* generated eukaryotic transcriptomes

**Joanna Moreton^1, 2^*, Abril Izquierdo^2^ and Richard D. Emes^1, 2^**

*** Correspondence:** Corresponding Author: Joanna.Moreton@nottingham.ac.uk

# Supplementary Table

**Supplementary Table 1: List of tools described in the manuscript.** The tools are categorised according to type and are listed in alphabetical order for each category. A brief description and reference is provided for each tool.

| **Type** | **Tool** | **Brief description** | **Reference** |
| --- | --- | --- | --- |
| **Aligner** | TopHat2 | Splice-aware aligner of RNA-seq reads. | (Kim et al., 2013) |
| **Assembler** | Cufflinks | Transcriptome assembler which uses, as input, reads that have been aligned to a reference genome. It also estimates transcript abundance. | (Trapnell et al., 2010) |
|  | MIRA | *De novo* transcriptome assembler of RNA-seq data. | (Chevreux et al., 2004) |
|  | Oases | *De novo* transcriptome assembler of RNA-seq data. | (Schulz et al., 2012) |
|  | Scripture | Transcriptome assembler which uses, as input, reads that have been aligned to a reference genome. | (Guttman et al., 2010) |
|  | Trans-ABySS | *De novo* transcriptome assembler of RNA-seq data. | (Robertson et al., 2010) |
|  | Trinity | *De novo* transcriptome assembler of RNA-seq data. | (Grabherr et al., 2011) |
| **Differential expression analysis** | DESeq2 | Differential expression analysis of sequence read count data. | (Love et al., 2014) |
|  | EdgeR | Differential expression analysis of sequence read count data. | (Robinson et al., 2010) |
| **Assessment** | BUSCO | Tool to assess assembly and annotation completeness using sets of Benchmarking Universal Single-Copy Orthologs. | (Simão et al., 2015) |
|  | CEGMA | Discontinued tool which was used to search a dataset for a set core genes conserved across a wide range of eukaryotic species. | (Parra et al., 2007) |
|  | DETONATE | Software package to assess *de novo* transcriptome assemblies. It includes both a reference-free (RSEM-EVAL) and reference-based (REF-EVAL) method. | (Li et al., 2014) |
|  | TransRate | Reference-free evaluation tool for *de novo* transcriptome assemblies. | (Smith-Unna et al., 2015) |
| **Annotation** | HMMER3 | Tool to search sequence databases to identify homologous sequences. | (Finn et al., 2011) |
|  | InterProScan | Software package which scans sequences against protein signatures in the InterPro member databases. | (Zdobnov and Apweiler, 2001) |
|  | RepeatMasker | Program to search for repeats and low complexity sequences. | repeatmasker.org |
|  | Tandem Repeats Finder | Tool which finds and displays tandem repeats in sequences | (Benson, 1999) |
|  | Trinotate | Pipeline for annotating transcriptomes especially *de novo* assemblies. | trinotate.github.io |
| **Availability** | afterParty | Web server and downloadable tool to make transcriptome data publicly available. It can also assemble 454 reads and annotate contigs. | (Jones and Blaxter, 2013) |
|  | CBrowse | Web browser for visualising and analysing transcriptome assemblies. | (Li et al., 2012) |
|  | RNAbrowse | Package with a web interface that can be used to store and visualise *de novo* transcriptome data. | (Mariette et al., 2014) |
|  | SRA | The Sequence Read Archive (SRA) is a public repository of raw data from next-generation sequencing platforms. It is available at the NCBI, EBI and DDBJ. | (Kodama et al., 2012) |
| **Other** | BioMart | Provides an interface to many databases. It includes tools for data analysis and visualisation. | (Smedley et al., 2015) |
|  | BLAST | Sequence similarity tool that can be used to compare query sequences to sequence databases. | (McGinnis and Madden, 2004) |
|  | BLASTX | Sequence similarity tool to compare translated nucleotide query sequences to protein databases. | (Altschul et al., 1997) |
|  | JBrowse | Web-based genome browser. | (Skinner et al., 2009) |
|  | TRUFA | Web server for the analysis of RNA-seq data. | (Kornobis et al., 2015) |

Altschul, S.F., Madden, T.L., Schaffer, A.A., Zhang, J., Zhang, Z., Miller, W., and Lipman, D.J. (1997). Gapped BLAST and PSI-BLAST: a new generation of protein database search programs. *Nucleic Acids Res* 25**,** 3389-3402.

Benson, G. (1999). Tandem repeats finder: a program to analyze DNA sequences. *Nucleic Acids Research* 27**,** 573-580. doi: 10.1093/nar/27.2.573.

Chevreux, B., Pfisterer, T., Drescher, B., Driesel, A.J., Müller, W.E., Wetter, T., and Suhai, S. (2004). Using the miraEST assembler for reliable and automated mRNA transcript assembly and SNP detection in sequenced ESTs. *Genome research* 14**,** 1147-1159.

Finn, R.D., Clements, J., and Eddy, S.R. (2011). HMMER web server: interactive sequence similarity searching. *Nucleic acids research***,** gkr367.

Grabherr, M.G., Haas, B.J., Yassour, M., Levin, J.Z., Thompson, D.A., Amit, I., Adiconis, X., Fan, L., Raychowdhury, R., Zeng, Q., Chen, Z., Mauceli, E., Hacohen, N., Gnirke, A., Rhind, N., Di Palma, F., Birren, B.W., Nusbaum, C., Lindblad-Toh, K., Friedman, N., and Regev, A. (2011). Full-length transcriptome assembly from RNA-Seq data without a reference genome. *Nat Biotechnol* 29**,** 644-652. doi: 10.1038/nbt.1883.

Guttman, M., Garber, M., Levin, J.Z., Donaghey, J., Robinson, J., Adiconis, X., Fan, L., Koziol, M.J., Gnirke, A., and Nusbaum, C. (2010). Ab initio reconstruction of cell type-specific transcriptomes in mouse reveals the conserved multi-exonic structure of lincRNAs. *Nature biotechnology* 28**,** 503-510.

Jones, M., and Blaxter, M. (2013). afterParty: turning raw transcriptomes into permanent resources. *BMC bioinformatics* 14**,** 301.

Kim, D., Pertea, G., Trapnell, C., Pimentel, H., Kelley, R., and Salzberg, S.L. (2013). TopHat2: accurate alignment of transcriptomes in the presence of insertions, deletions and gene fusions. *Genome Biol* 14**,** R36.

Kodama, Y., Shumway, M., and Leinonen, R. (2012). The Sequence Read Archive: explosive growth of sequencing data. *Nucleic acids research* 40**,** D54-D56.

Kornobis, E., Cabellos, L., Aguilar, F., Frías-López, C., Rozas, J., Marco, J., and Zardoya, R. (2015). TRUFA: A User-Friendly Web Server for de novo RNA-seq Analysis Using Cluster Computing. *Evolutionary Bioinformatics Online* 11**,** 97-104. doi: 10.4137/EBO.S23873.

Li, B., Fillmore, N., Bai, Y., Collins, M., Thomson, J.A., Stewart, R., and Dewey, C.N. (2014). Evaluation of de novo transcriptome assemblies from RNA-Seq data. *Genome biology* 15**,** 553.

Li, P., Ji, G., Dong, M., Schmidt, E., Lenox, D., Chen, L., Liu, Q., Liu, L., Zhang, J., and Liang, C. (2012). CBrowse: a SAM/BAM-based contig browser for transcriptome assembly visualization and analysis. *Bioinformatics* 28**,** 2382-2384. doi: 10.1093/bioinformatics/bts443.

Love, M.I., Huber, W., and Anders, S. (2014). Moderated estimation of fold change and dispersion for RNA-seq data with DESeq2. *Genome Biol* 15**,** 550.

Mariette, J., Noirot, C., Nabihoudine, I., Bardou, P., Hoede, C., Djari, A., Cabau, C., and Klopp, C. (2014). RNAbrowse: RNA-Seq de novo assembly results browser. *PLoS One* 9**,** e96821. doi: 10.1371/journal.pone.0096821.

Mcginnis, S., and Madden, T.L. (2004). BLAST: at the core of a powerful and diverse set of sequence analysis tools. *Nucleic Acids Res* 32**,** W20-25. doi: 10.1093/nar/gkh435.

Parra, G., Bradnam, K., and Korf, I. (2007). CEGMA: a pipeline to accurately annotate core genes in eukaryotic genomes. *Bioinformatics* 23**,** 1061-1067. doi: 10.1093/bioinformatics/btm071.

Robertson, G., Schein, J., Chiu, R., Corbett, R., Field, M., Jackman, S.D., Mungall, K., Lee, S., Okada, H.M., Qian, J.Q., Griffith, M., Raymond, A., Thiessen, N., Cezard, T., Butterfield, Y.S., Newsome, R., Chan, S.K., She, R., Varhol, R., Kamoh, B., Prabhu, A.L., Tam, A., Zhao, Y., Moore, R.A., Hirst, M., Marra, M.A., Jones, S.J., Hoodless, P.A., and Birol, I. (2010). De novo assembly and analysis of RNA-seq data. *Nat Methods* 7**,** 909-912. doi: 10.1038/nmeth.1517.

Robinson, M.D., Mccarthy, D.J., and Smyth, G.K. (2010). edgeR: a Bioconductor package for differential expression analysis of digital gene expression data. *Bioinformatics* 26**,** 139-140.

Schulz, M.H., Zerbino, D.R., Vingron, M., and Birney, E. (2012). Oases: robust de novo RNA-seq assembly across the dynamic range of expression levels. *Bioinformatics* 28**,** 1086-1092.

Simão, F.A., Waterhouse, R.M., Ioannidis, P., Kriventseva, E.V., and Zdobnov, E.M. (2015). BUSCO: assessing genome assembly and annotation completeness with single-copy orthologs. *Bioinformatics*. doi: 10.1093/bioinformatics/btv351.

Skinner, M.E., Uzilov, A.V., Stein, L.D., Mungall, C.J., and Holmes, I.H. (2009). JBrowse: a next-generation genome browser. *Genome Res* 19**,** 1630-1638. doi: 10.1101/gr.094607.109.

Smedley, D., Haider, S., Durinck, S., Pandini, L., Provero, P., Allen, J., Arnaiz, O., Awedh, M.H., Baldock, R., and Barbiera, G. (2015). The BioMart community portal: an innovative alternative to large, centralized data repositories. *Nucleic acids research***,** gkv350.

Smith-Unna, R.D., Boursnell, C., Patro, R., Hibberd, J.M., and Kelly, S. (2015). TransRate: reference free quality assessment of de-novo transcriptome assemblies. *bioRxiv***,** 021626.

Trapnell, C., Williams, B.A., Pertea, G., Mortazavi, A., Kwan, G., Van Baren, M.J., Salzberg, S.L., Wold, B.J., and Pachter, L. (2010). Transcript assembly and quantification by RNA-Seq reveals unannotated transcripts and isoform switching during cell differentiation. *Nat Biotechnol* 28**,** 511-515. doi: 10.1038/nbt.1621.

Zdobnov, E.M., and Apweiler, R. (2001). InterProScan – an integration platform for the signature-recognition methods in InterPro. *Bioinformatics* 17**,** 847-848. doi: 10.1093/bioinformatics/17.9.847.
